# Supplementary material for: A century of change in the California Current: upwelling system amplifies acidification
Source: Nat Commun. 2025 Nov 13;16:9661. doi: 10.1038/s41467-025-63207-6 (PMC12615725; doi:10.1038/s41467-025-63207-6)
Supplement: Supplementary file 1 — Supplementary Information [file 41467_2025_63207_MOESM1_ESM.pdf]

## Supplementary Information Outline


**Supplementary Methods**

1. Coral Calibration and Data Preparation
    - 1.1 Averaging coral data across locations in the Salish Sea
    - 1.2 Seasonal cycles of carbonate chemistry in the Salish Sea to inform observational-coral offset
    - 1.3 Applying the observation-coral pH offset and summertime growth of *Balanophyllia elegans*
    - 1.4 Inferring unknown information about West Coast Corals

**Supplementary Discussion**

1. Coral Record Interpretation and Comparison to Model and Other Proxy Estimates
    - 1.1 Process-based arguments to assess 20th century acidification in the Salish Sea
    - 1.2 Comparison between Salish Sea box model and coral-based $\Delta pCO_2$
    - 1.3 Comparison between West Coast model and coral records of historic $pCO_2$
    - 1.4 Reconstruction of fossil foraminifera pH and $pCO_2$ based on *G. bulloides* shell weights
2. Independent Records of Comparison and Climate Factors
    - 2.1 Impact of the Pacific Decadal Oscillation and climate variability on 20th century acidification

**Supplementary Notes**

1. Background Information on Study Area
    - 1.1 The California Current upwelling system
    - 1.2 Characterizing the carbonate chemistry in the Salish Sea

**Supplementary Figures**

Supplementary Fig. 1 Map of modern and historic coral locations
Supplementary Fig. 2 Raw and location-averaged $\delta^{11}B$ data (Salish Sea and West Coast)
Supplementary Fig. 3 pH climatologies in the Salish Sea and CCS
Supplementary Fig. 4 pH vs DIC plot in the Salish Sea
Supplementary Fig. 5 Schematic of steady state box model in the Salish Sea box model
Supplementary Fig. 6 General patterns of carbonate chemistry parameters in modern model
Supplementary Fig. 7 General patterns of modeled acidification over the 20th century
Supplementary Fig. 8 Profile of summertime $\Delta pCO_2$ in the CCS
Supplementary Fig. 9 ΔDIC attributed to 20th century changes in remineralization in the CCS
Supplementary Fig. 10 ΔDIC attributed to anthropogenic carbon and thermodynamic buffering effects in the CCS
Supplementary Fig. 11 Comparison between West Coast historic model and coral $pCO_2$
Supplementary Fig. 12 Reconstruction of fossil foraminifera record in the Santa Barbara Basin, California
Supplementary Fig. 13 Relationships between salinity, total alkalinity, and DIC at Admiralty Inlet
Supplementary Fig. 14 Reconstruction of fossil foraminifera record in the Santa Barbara

**Supplementary Tables**

Supplementary Table 1 Annual and summertime averages of DIC, TA, temperature, salinity, and pH at Admiralty Inlet
Supplementary Table 2 Annual and summertime observation-coral pH offsets
Supplementary Table 3 West Coast coral estimates of pH and $p\mathrm{CO_2}$
Supplementary Table 4 Average PDO Index for historic and modern time periods

**Supplementary References**

## Supplementary Methods

### 1. Coral Calibration and Data Preparation

Historic samples of *Balanophyllia elegans* (*B. elegans)* were collected by the R/V *Albatross* between 1888 and 1894, and by Ernest Harrison Quayle in 1932. All historic samples were archived in the Smithsonian National Museum of Natural History (Supplementary Fig. 1). In the Salish Sea, modern corals were collected from nearby locations in 2020 (Supplementary Fig. 1). The raw and location-averaged skeletal $\delta^{11}B$ data can be found in Supplementary Fig. 2. Skeletal $\delta^{11}B$ data can also be found in Supplementary Data.

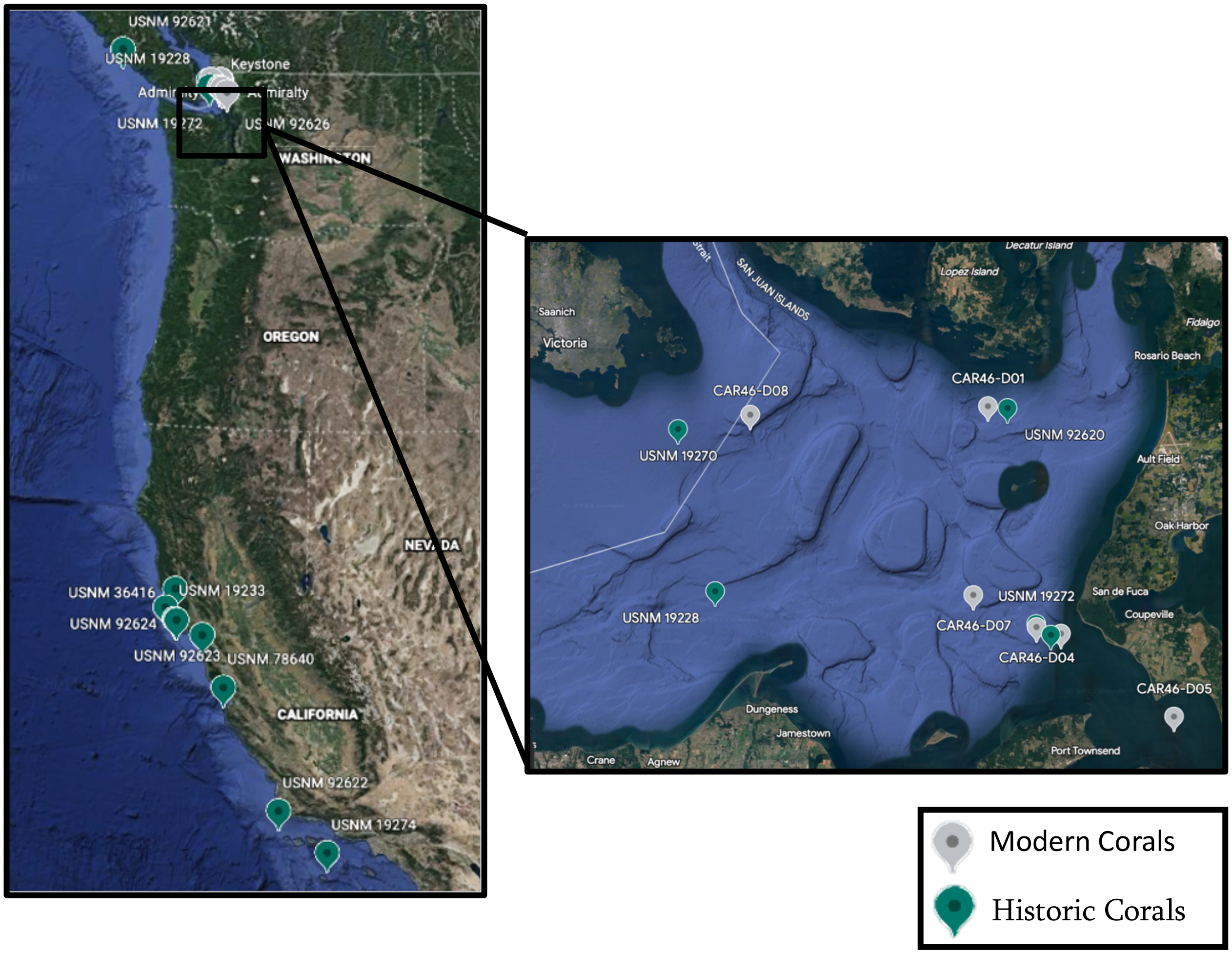


**Supplementary Fig. 1:** Map of historic and modern coral locations along the US West Coast (left) and the Salish Sea (right). Historic corals were collected between 1888 and 1894 with the exception of USNM 78638 and 78640 (1930s) and archived in the Smithsonian National Museum of Natural History (green). Modern corals were collected from nearby locations in the Salish Sea aboard the University of Washington R/V *Rachel Carson* in 2020 (gray). Location ID's specified in Supplementary Data.

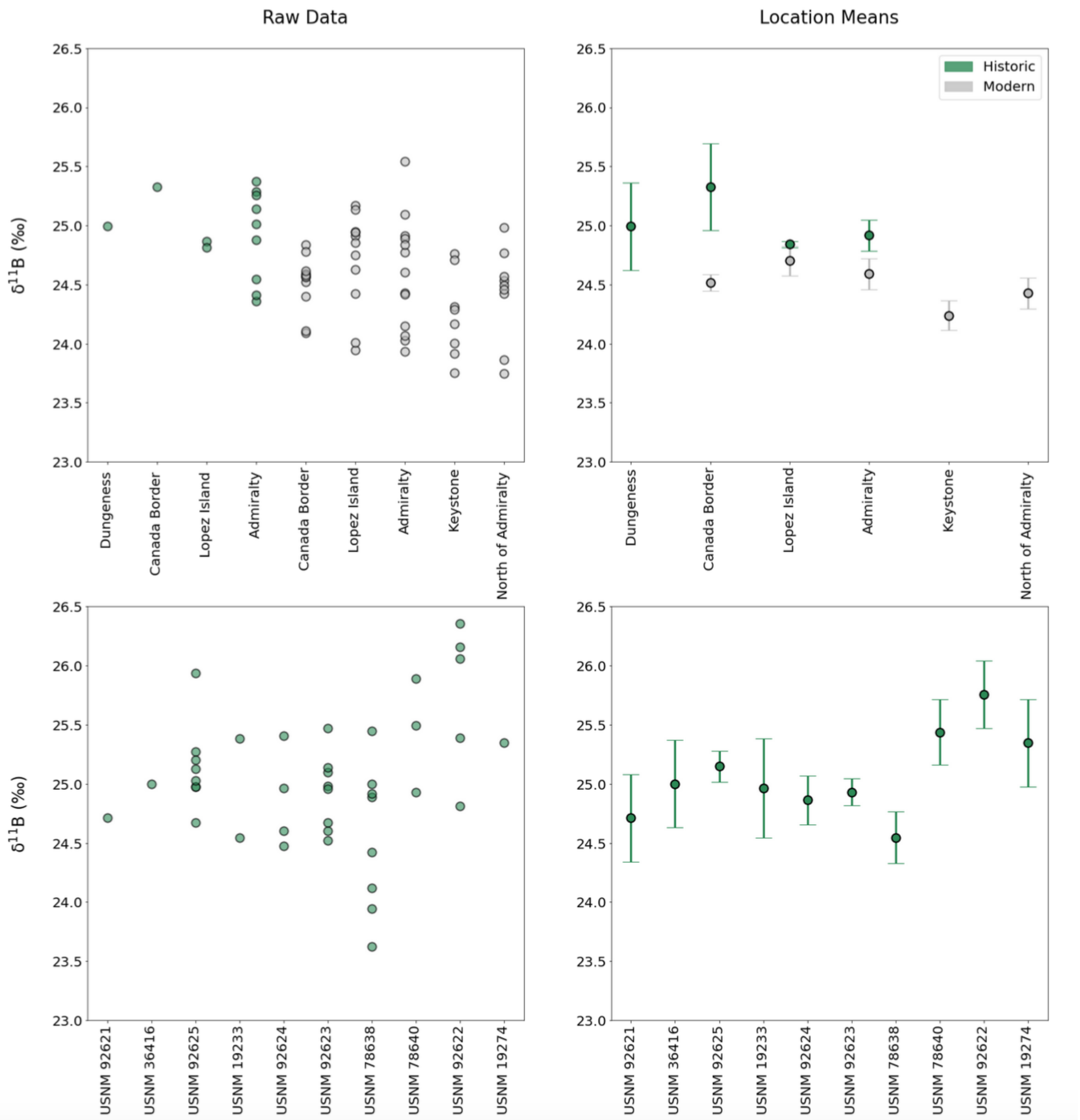


**Supplementary Fig. 2:** Skeletal $\delta^{11}B$ data from the Salish Sea (top row) and the US West Coast (bottom row). Left column: Raw skeletal $\delta^{11}B$ at individual locations. Each point represents a sample from a single coral. Historic measurements are shown in green and modern measurements shown in gray. Historic Salish Sea $n = 13$; modern Salish Sea $n = 52$; historic West Coast $n = 41$. Right column: Location-averaged $\delta^{11}B$ data. Error bars represent the standard error of the mean at each location. If only one coral was sampled, the population standard deviation was used. The population standard deviation was calculated by averaging the sample standard deviation at every location where 8 or more corals were collected. USNM 78638 was excluded from this calculation due to the high variability associated with tide pools.

Prior to interpreting the signals implied by the coral records, several steps are taken to prepare the data for analysis. First, we consider whether the coral data in the Salish Sea are representative of the whole basin or are location specific (Supplementary Methods 1.1). Modern coral data in the Salish Sea are then compared with modern bottle data to determine if corals in the wild match culture-based calibrations and assess the need for an observation-coral offset (Supplementary Methods 1.2). Application of the observation-coral offset and assumptions regarding the coral metadata are described in Supplementary Methods 1.3 and 1.4, respectively.

## 1.1 Averaging coral data across locations in the Salish Sea

Historic and modern coral data from the Salish Sea span multiple locations and depths (Supplementary Data, Supplementary Fig. 1). Given the spatial distribution, we consider whether the data are representative of the whole Salish Sea or are location specific. To evaluate the similarities and differences among locations, carbonate chemistry is analyzed from the Live Ocean model (https://faculty.washington.edu/pmacc/LO/LiveOcean.html), a product from the University of Washington Coastal Modeling Group led by Dr. Parker MacCready that simulates seawater properties in the Salish Sea. Parameters including DIC, TA, salinity, and temperature are analyzed over three years (2018–2020) and analysis of variance (ANOVA) tests are performed to assess the difference in means at each location. A Tukey Honestly Significant Difference test is then used to determine which locations are statistically different from each other. Across parameters, the locations near Admiralty Inlet are statistically indistinguishable, excluding the modern coral location near Keystone Jetty, which does not correspond to a historic coral location anyway. Admiralty Inlet locations are statistically different from those in the Strait of Juan de Fuca, as makes sense due to path of circulation.

We average the historic and modern coral data together due to the clustering of coral collection locations near Admiralty Inlet, meant to represent a central location in the Salish Sea. A statistical test of modern coral data also revealed no significant difference between corals collected near Admiralty Inlet from those in the Strait of Juan de Fuca. The extra detail gained from analyses separated by location does not add new insights to 20$^{th}$ century acidification in the Salish Sea. Including historic and modern coral data outside of Admiralty Inlet in the analysis does not change the ultimate conclusions regarding 20$^{th}$ century acidification.

## 1.2 Seasonal cycles of carbonate chemistry in the Salish Sea to inform observation-coral offset

Upon averaging the coral data across locations in the Salish Sea, the modern coral data are compared to modern observational data to determine if modern coral data in the wild match culture-based calibrations. To align the coral and observational records, we establish the observation-coral offset with data from Admiralty Inlet due to the abundance of both modern coral data and bottle data. Observational data (pH, DIC, TA, temperature and salinity) were collected from Admiralty Inlet (Washington Ocean Acidification Center (WOAC) cruise station 21), sampled on cruises between 2008 and 2018. While this dataset provides a glimpse into annual averages of carbonate chemistry parameters, it is necessary to fit polynomial curves to the data to estimate average conditions across seasons due to the sparsity of samples. The parameter averages obtained from these climatological curves produce the values to which we compare the coral data and calculate the observation-coral offset.

Seasonal cycles for DIC, TA, temperature, and salinity are estimated from WOAC station 21 bottle data below 50 meters depth, the same depth of coral collection at Admiralty Inlet (Supplementary Data). Upon capturing an annual snapshot of parameters at >50 meters depth at Admiralty Inlet, third order polynomials are fitted to the DIC, TA, and salinity data. A polynomial fit is chosen to capture the non-periodic nature of the seasonal cycles. Annual and summer (June-August) averages of each parameter are calculated from the seasonal fitted curves (Supplementary Table 1). The shapes of the seasonal cycles are confirmed by modeled three-year time series from the LiveOcean model at the same location and depth. While the tails of the polynomial fits include extreme values in some cases, they do not appreciably impact the summer or annual averages. To confirm, we also calculated a summer average informed by WOAC station 21 bottle data at >50 meters depth from July only (Supplementary Table 1). The July data average aligns with parameter estimates obtained from the summertime seasonal fitted curves. The average pH values obtained from the Admiralty Inlet seasonal cycles form the foundation on which we base the observation-coral pH offset.

**Supplementary Table 1:** The top two rows represent annual and summer averages of pH, TA (µmol/kg), DIC (µmol/kg), temperature (°C), and salinity (PSU) at >50 meters depth at Admiralty Inlet in the Salish Sea. Values represent the annual and summertime means of the seasonal cycle fits estimated from WOAC bottle data. The bottom row represents summer averages of pH, TA, DIC, temperature, and salinity at >50 meters depth informed by WOAC bottle data from July only.

| | **DIC** (µmol/kg) | **pH** (total) | **TA** (µmol/kg) | **Temp** (°C) | **Sal** (PSU) |
|---|---|---|---|---|---|
| **Seasonal Fitted Curve (Annual Average)** | 2116 | 7.73 | 2165 | 8.82 | 31.9 |
| **Seasonal Fitted Curve (Summer Average)** | 2120 | 7.75 | 2177 | 8.84 | 32.1 |
| **July Data Only (Summer Average)** | 2106 | 7.75 | 2165 | 9.6 | 31.8 |

1.3 Applying the observation-coral pH offset and summertime growth of *Balanophyllia elegans*

As described in Supplementary Methods 1.2, the observation-coral pH offset is determined by comparing the annual and summertime averages of bottle data to modern coral data at Admiralty Inlet in the Salish Sea. The averages obtained from observational data are described in Supplementary Methods 1.2 and can be found in Supplementary Table 1. The average coral-based pH is determined from the skeletal $\delta^{11}$B-seawater pH relationship determined from a controlled laboratory experiment[1]. The summertime average of DIC from observational data (Supplementary Methods 1.2) is used to obtain a $\delta^{11}$B DIC-correction value (Methods, Equation 1). The $\delta^{11}$B DIC-correction is then substituted into the *B. elegans* calibration equation to calculate a coral-based pH estimate of 7.85 (SE = 0.013) (Methods, Equation 2). pH offsets of -0.127 (annual offset) and -0.101 (summertime offset) are attained by calculating the difference between the coral-based pH estimate and the observational pH data (Supplementary Table 2).

**Supplementary Table 2:** Annual and summer pH offsets between observational data and coral data (observational average – coral average). Annual observational pH average is from a fitted seasonal cycle curve informed by WOAC station 21 bottle data at >50 meters to match the coral depth of collection. Summer observational pH average is obtained from WOAC station 21 bottle data at >50 meters from July only. The coral-based pH average is calculated from modern data at locations near Admiralty Inlet.

| Summer pH Offset | Annual pH Offset |
|---|---|
| -0.101 | -0.127 |

We believe the laboratory-derived $\delta^{11}$B-pH calibration requires an offset when applied to wild corals due to seasonal growth biases not replicated in the lab experiments. Several studies suggest that some coral species exhibit growth biases in the summer and do not capture an annual, mean state of surrounding seawater. For instance, Fadlallah 1983[2] discovered that food availability, as a result of upwelling, could promote growth and larval settlement of *B. elegans*. Jantzen et al. 2013[3] also found that *Desmophyllum dianthus*, another species of cold-water coral, exhibit higher growth rates in the summer compared to the winter. Beck et al. 2018[4] confirmed these seasonal growth patterns in *D. dianthus* and determined that the high summer growth rates are likely due to seasonal differences in food availability during peak upwelling of nutrient-rich waters. In addition, the reproduction cycle of *D. diantus* typically occurs in August and may also explain the reduced growth rates in winter due to the redirection of energy towards reproduction.

In the Salish Sea, the observation-coral pH offset assuming annual versus summer averages is within 0.03 pH units and has little influence on conclusions about 20$^{th}$ century acidification (Supplementary Table 2). The similarity between the summer and annual offsets is demonstrated in the pH climatology, where the summer data span the inflection point and are similar in magnitude to the annual average (Supplementary Fig. 3). To calibrate the coral records in the wild, we apply the summer pH offset = -0.101 to all pH data produced by the coral record.

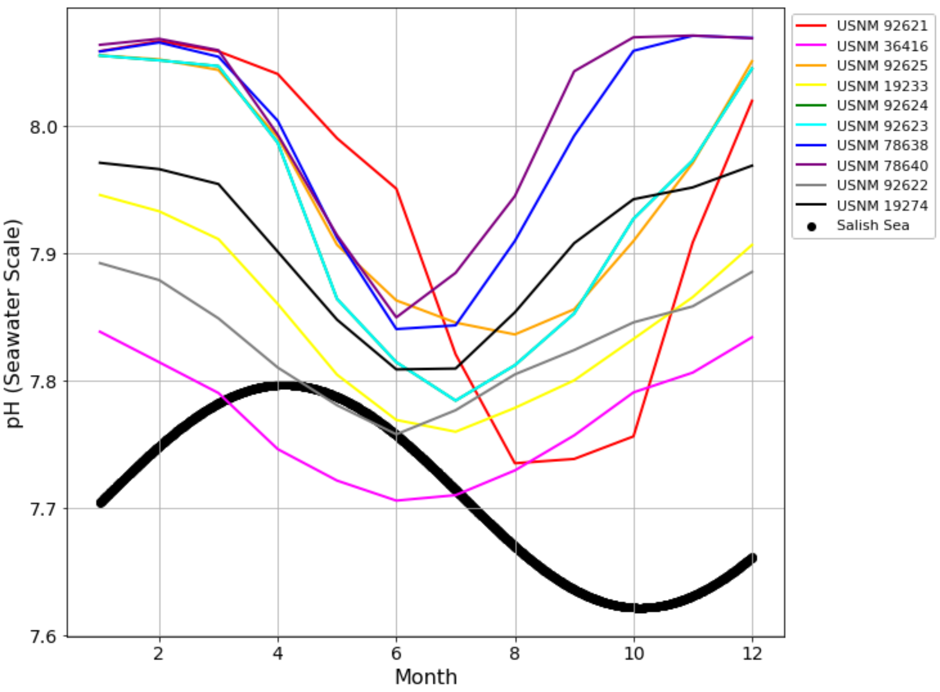


**Supplementary Fig. 3:** pH climatologies from the West Coast and Salish Sea coral locations. The Salish Sea seasonal cycle is constructed from WOAC bottle data and a curve fitting method at Admiralty Inlet (Supplementary Methods 1.2). West Coast pH climatologies are calculated from a 7-year modeled average in a Regional Ocean Modeling System (ROMS, Methods) from November 2000-December 2007. While Admiralty Inlet experiences lowest pH in the fall, the West Coast experiences lowest pH in the summer, generally from June to August. The delay in acidified conditions in the northern CCS is also apparent in the lagged pH decline at our northernmost West Coast location, near the mouth of the Salish Sea (USNM 92621). This is likely due to the accumulation of respiration and acidified conditions through the upwelling season at these locations where there is longer retention. While the annual pH average at Admiralty Inlet is approximately equal to the summer pH average, the annual and summer pH averages along the West Coast diverge. Since the corals are presumed to grow preferentially in the summer during the upwelling season, the observation-coral pH offset is established from observational summer averages (Supplementary Table 1). Therefore, the historic West Coast coral data are compared to modeled summer averages.

While the summer and annual pH averages are approximately equivalent in the Salish Sea, locations along the West Coast have different climatologies. Supplementary Fig. 3 shows pH climatologies from the Salish Sea and every coral location in the CCS. The pH seasonal cycle from the Salish Sea is informed by WOAC bottle data, whereas the pH climatologies along the West Coast represent 7-year Regional Ocean Modeling System (ROMS) output averages from December 2000–November 2007. The West Coast locations experience low-pH values earlier in the season compared to the Salish Sea. As a result, the annual and summer averages along the West Coast differ whereas they are approximately equivalent in the Salish Sea. To align the historic West Coast coral record, we assume that the corals also capture a summer average of surrounding seawater conditions.

Alignment of the modern observational record and modern coral record allows us to obtain absolute values of pH. While much can be gleaned from the relative pH difference produced by historic and modern coral pairs in the Salish Sea, determining a pH offset applicable to all wild corals allows us to anchor the West Coast $\delta^{11}B$ data, where repeat occupations to collect corals in the modern era have not been achieved.

1.4 Inferring unknown information about West Coast coral locations

Of the ten coral locations along the outer West Coast, eight of the sites were collected by the R/V *Albatross I*. Existing records from the expedition contain pertinent information including latitude, longitude, and depth of collection (Supplementary Data). The two remaining coral sites, USNM 78638 and USNM 78640, were collected by Ernest Harrison Quayle (1891–1956). While the general locations of the sites were recorded, information including depth, latitude, and longitude are unknown. Only USNM 78640 has a year of collection in 1932.

Due to the lack of information, several inferences are made. First, it is presumed that USNM 78638 was also sampled around 1932. After embarking on the Whitney South Sea Expedition until 1922, Quayle returned to the US with a keen interest in coral reefs. Quayle moved to California in 1931 at the peak of his coral research and published an article on fossil corals in the Transactions of the San Diego Society of Natural History in 1932 (https://data.library.amnh.org/archives-authorities/id/amnhp_1002468). Therefore, we assume that USNM 78638, located near San Francisco, California, was also collected in the early 1930s. Second, the depths of collection for both USNM 78638 and USNM 78640 are presumed to be ~25 meters. USNM 78638 was reportedly collected at a beach and a vessel was not likely used. Third, latitudes and longitudes for both locations were chosen close to the coast given the probable shore-based collection method. Lastly, although the corals were collected in the 1930s, we still compared the coral data to the 1891–1904 ROMS output since the vast majority of anthropogenic $CO_2$ was emitted later in the $20^{th}$ century.

The corals at site USNM 78638 were collected from a tidepool near Moss Beach, California–a highly variable environment. This was the only location where modern $pCO_2$ appeared lower than historic $pCO_2$, likely due to the extreme variability of the intertidal zone that influenced the $\delta^{11}B$ skeletal signatures. Given this high natural variability, long-term signals can be obscured by environmental noise. Consequently, these data were not heavily weighted in our analysis or conclusions. Had they been central to our study, additional efforts to constrain collection dates and reduce uncertainty would have been undertaken.

## Supplementary Discussion

### 1. Coral Record Interpretation and Comparison to Model and Other Proxy Estimates

The coral records in both the Salish Sea and the outer West Coast suggest that the CCS has experienced amplified acidification over the $20^{th}$ century, exceeding the rise in atmospheric $CO_2$. We contextualize the coral records with several independent sources to determine the main driver of the acidification signal.

In the Salish Sea, our historic-modern coral pairs imply that pH decreased -0.095 units (standard error of the difference between means $\sigma_{M_1-M_2}$ = 0.025) and $p$CO$_2$ increased 172 +/- 41 µatm over the industrial era. In Supplementary Discussion 1.1, we invoke process-based arguments to assess the likelihood of various mechanisms to explain the coral data. We also compare the coral record with a box model of the Salish Sea, for which a ROMS model sets the composition of upwelled water entering the box model, to consider the impact of estuarine processes on the 20$^{th}$ century signal (Supplementary Discussion 1.2).

Along the outer West Coast, we directly compare coral estimates with ROMS, which resolves the coastal CCS. Historic model summertime averages of $p$CO$_2$ are compared to historic coral $p$CO$_2$ at every location. The corals demonstrate that the CCS has experienced amplified acidification over the industrial era, and even capture the trend of more severe amplified acidification with depth. In Supplementary Discussion 1.3, we describe the alignment between the coral- and model-based estimates of 20$^{th}$ century acidification. While the corals generally reproduce modeled predictions, the Santa Barbara Basin locations diverge most from the model and correspond to a much lower historic $p$CO$_2$ than that presented in the model. Supplementary Discussion 1.4 describes a previously published proxy record of carbonate chemistry in the Santa Barbara Basin that also indicates a larger magnitude of 20$^{th}$ century acidification.

## 1.1 Process-based arguments to assess 20$^{th}$ century acidification in the Salish Sea

As described in Supplementary Notes 1.2, several processes influence carbonate chemistry in the Salish Sea. Supplementary Fig. 4 captures the implied changes in carbonate chemistry over the 20$^{th}$ century produced by the coral data assuming the dominant mechanism is 1) calcification/dissolution, 2) mixing source waters, 3) shifts in biological activity, and 4) $CO_2$ invasion. Below, we discuss the likelihood of each process serving as the driver of 20$^{th}$ century acidification.

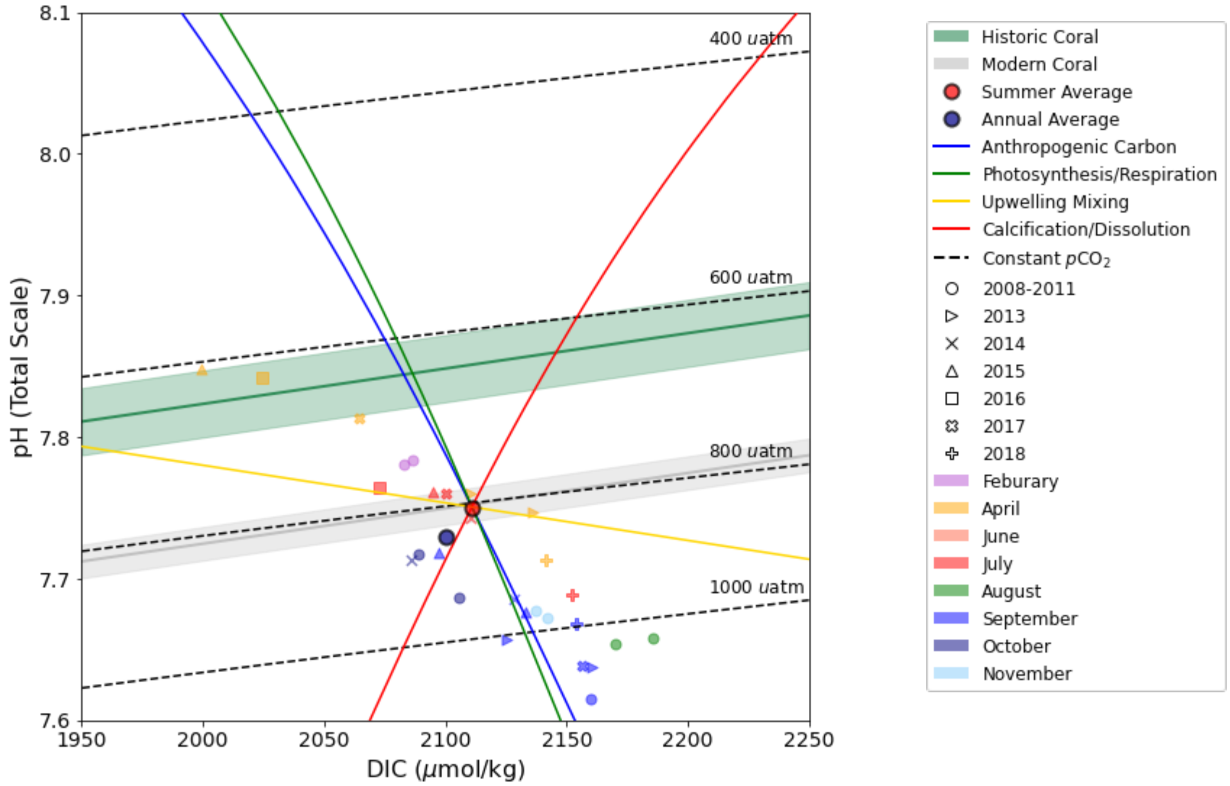


**Supplementary Fig. 4:** 20th century changes in $pCO_2$ ($\Delta pCO_2$) and pH ($\Delta$pH) in the Salish Sea. Historic (green) and modern (gray) coral records averaged across locations in the Salish Sea (Equations 1 and 2). Changes in seawater chemistry over the past century are bounded by these two lines. Error bands represent standard errors of the mean. Black dashed contours represent lines of constant $pCO_2$. Note that contours of $pCO_2$ and the coral-derived bounds on $pCO_2$ are roughly parallel, so the coral data act to constrain $pCO_2$ tightly without additional information. The coral record indicates that $pCO_2$ increased by 172 µatm between the 1890s and 2020 (± 41 µatm standard error of the difference between means $\sigma_{M_1-M_2}$). Colored markers represent observational data from 70 meters depth at the Washington Ocean Acidification Center (WOAC) station 21[5]. The seasonality in observational data is primarily driven by mixing and freshwater inputs. Summer average (JJA) seawater properties from WOAC station 21 are represented by the red dot: pH = 7.75, DIC = 2106 µmol/kg, TA = 2165 µmol/kg (Supplementary Table 1). To align the modern coral record with the observational data summer average, a pH offset of -0.10 has been applied to both the historic and modern coral records. Colored lines represent the ways that key biogeochemical processes affect pH and DIC: Seawater $CO_2$ invasion (blue), primary production and respiration (green), upwelling and mixing source waters (yellow), and calcium carbonate formation and dissolution (red) (Supplementary Discussion 1.1). Assuming anthropogenic carbon is the main driver of the 20th century change, we calculate changes in pH and DIC from the intersections of the seawater $CO_2$ invasion line and historic and modern coral records. The coral data estimate $\Delta$pH = -0.095 (± 0.025 standard error of the difference between means $\sigma_{M_{Modern}-M_{Historic}}$) and $\Delta$DIC = 28 µmol/kg for the bottom waters of the Salish Sea.

*Shift in calcium carbonate formation and dissolution rates*
The formation of calcium carbonate describes the process by which marine organisms build their shells from calcium ion and carbonate ions in seawater. Calcium carbonate formation implies consumption of 2 μmol/kg TA for every 1 μmol/kg DIC, resulting in increased $pCO_2$ and acidity. The coral records support a decrease in pH, which indicates that calcium carbonate formation would have had to increase to explain the observed pH change. However, evidence suggests that calcification actually declines in response to corrosive conditions ($\Omega_{aragonite}<1$) that accompany pH levels <7.75 in the CCS[6,7]. Assuming the physiology of calcification has not changed, it is unlikely that shifts in calcification explain the 20th century change in pH implied by the coral records given these two effects oppose one other.

*Shift in source water mixing*
We also consider whether the coral-based ΔpH can be attributed to a shift in the mixing ratio between oceanic and riverine sources. As discussed in Supplementary Notes 1.2, variation in Salish Sea carbonate chemistry is strongly influenced by water mass mixing. This foundational understanding of seasonal variability today provides an important constraint to assess the likelihood of shifting seasonal patterns as the dominant driver of 20th century change. As seen in Supplementary Fig. 4, seasonal variability in the modern, observational pH data is on the order of ΔpH predicted by the corals; therefore, we explore the possibility of an amplified season to explain 20th century acidification.

In general, observational pH data from the spring and summer months overlap with values implied by the historic coral record (Supplementary Fig. 4). If a shift in mixing (ie more freshwater influx in the historic era) were the main driver of 20th century acidification, then the mean state of the Salish Sea in the early 1900s would resemble modern-day spring and summer carbonate chemistry. As salinity in the Salish Sea is lower in the spring and summer, and salinity serves as a conservative parameter to trace water mass mixing, our shift in mixing hypothesis implies that river flow must have been larger in the early 20th century relative to today. Performing a two end-member mixing system of equations, the observational data suggests that river discharge must have declined by ~20% over the past century to explain the coral-based ΔpH. A best-fit linear regression was applied to Skagit River discharge data from 1940–2020 (USGS Skagit River gauge 1200500). The first three years of the 80-year time series were excluded from the linear regression due to an anomalously severe drought and several consecutive low-precipitation years that biased the slope (Washington State Governor's Report). This analysis suggests that discharge has declined by only ~5% over the past 130 years, extrapolated back in time to 1890.

This extrapolation likely underestimates changes in river discharge, as the river gauge was put in place after major dam construction on the Skagit River in the early 20th century. This same analysis was applied to another USGS River Gauge (12194000) that extends further back in time to 1925, however this analysis reveals that river discharge declined by ~6%. Thus, while dam construction likely influenced carbonate chemistry in the Admiralty Inlet area, the coral data suggests an unusually large shift in river discharge would have had to occur over the past 130 years to explain the ΔpH implied by the corals. While dam construction and river discharge likely influenced carbonate chemistry to some extent in the Salish Sea, they are not sufficient to explain the 20th century change in pH.

*Shift in primary production and biological activity*
The biological activity and $CO_2$ invasion process lines do not diverge substantially, indicating that the effects of these processes on ΔpH, ΔDIC, and $\Delta pCO_2$ are approximately equal. Only exceptional shifts in biological activity and a historic ocean with more respiration could have produced the magnitude of change observed in pH, $pCO_2$, and DIC produced by the corals. However, stable isotope analyses of marine-derived organic matter in the Salish Sea over the past century reveal no significant trend in biological activity[8]. Therefore, anthropogenic carbon is the driving force of 20th century acidification in the Salish Sea.

1.2 Comparison between Salish Sea box model and coral-based $\Delta pCO_2$

The process-based arguments described in Supplementary Discussion 1.1 provide useful constraints to evaluate the main driver of 20th century acidification. In addition to these lines of evidence, we compare the coral-based estimate of acidification to a simple steady-state box model representing the Salish Sea. Since our ROMS of the CCS does not resolve the Salish Sea, ROMS sets the composition of upwelled waters that enter the box model. The box model predicts both historic and modern DIC in the Salish Sea assuming only two processes, estuarine circulation of upwelled waters from the CCS and air-sea gas exchange. A schematic of the box model can be found in Supplementary Fig. 5.

As demonstrated in Supplement Equation (1), the steady-state simple box model is a function of:

$$\frac{dDIC}{dt} = \frac{1}{\tau}(DIC_{CCS} - DIC_{Salish\ Sea}) + \frac{p}{z}(K_0 \times pCO_{2\ atm} - pCO_{2\ Salish\ Sea}) \qquad \text{(S1)}$$

DIC of the inflowing, upwelled waters that enter the Salish Sea ($DIC_{CCS}$), DIC of the Salish Sea or outflowing water ($DIC_{Salish\ Sea}$), gas transfer velocity that governs air-sea gas exchange ($p$), the mixed layer depth ($z$), atmospheric $CO_2$ ($pCO_{2\ atm}$), Henry's Law equilibrium constant ($K_0$), the dissolved concentration of $CO_2$ in the Salish Sea ($pCO_{2\ Salish\ Sea}$), and residence time in the Salish Sea ($\tau$). Temperature, salinity, and total alkalinity are assumed to be constant in the outflowing and inflowing water masses and in both the historic and modern eras. Since $pCO_{2\ Salish\ Sea}$ is a function of $DIC_{Salish\ Sea}$ and total alkalinity, Supplementary Equation (1) can be solved for $DIC_{Salish\ Sea}$ alone under a steady-state scenario.

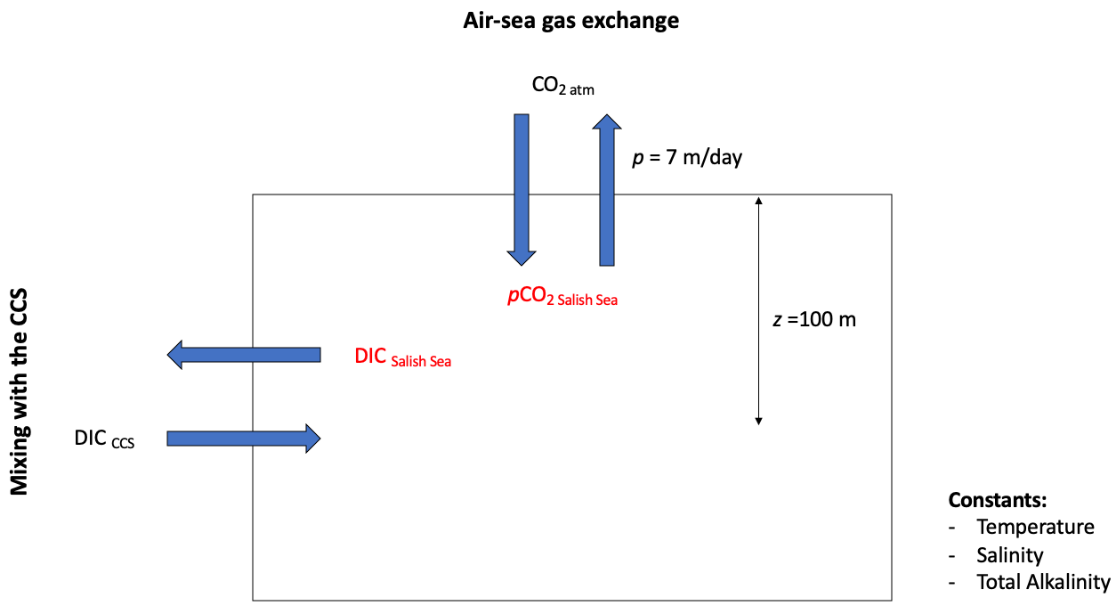


**Supplementary Fig. 5:** Schematic of the simple steady-state box model to calculate $\Delta pCO_2$ in the Salish Sea. We assume that carbonate chemistry in the Salish Sea is influenced by two processes including 1) exchange with upwelled waters from the CCS and 2) air-sea gas exchange. This simple box model is solved in both the historic and modern eras to produce an estimate of $\Delta pCO_2$. This estimate is then compared to the coral-based estimate of $\Delta pCO_2$ in the Salish Sea.

*$DIC_{CCS}$*:
Modeled DIC of the upwelled waters that enter the Salish Sea is obtained from ROMS for a 36 km x 36 km area at 150–200 meters depth at the mouth of the Strait of Juan de Fuca by calculating a 7-year summertime (JJA) average in both the historic and modern eras. While the historic timestamps of the coral and model records are offset by ~10 years, we assume the slow progression of $CO_2$ accumulation in the preindustrial era nullifies the need for a correction. To account for the modern temporal offset (corals collected 2020; model output ~2005), during which rapid carbon accumulation occurred, we advance the modeled $DIC_{CCS}$ from 2005 to 2020 to match the collection date of the modern corals and assume that this advance follows the known rise in atmospheric $CO_2$. Given that the water entering the Salish Sea from the CCS is 25 years old[9], the water mass represented in the modern model simulation was last in contact with the atmosphere in 1980. To augment modeled DIC from 2005 to 2020, we assume the waters entering the Salish Sea in 2020 were last in contact with the atmosphere in 1995 ($CO_{2\ atm}$ 1995 = 360 µatm; $\Delta CO_{2\ atm}$ 1980–1995 = 21 µatm). We quantify the impact of this anthropogenic carbon on DIC as described in Methods, Equations 3 and 4.

*$CO_{2\ atm}$*:
Atmospheric $CO_2$ was 295 and 415 $\mu$atm in the historic (1900) and modern (2020) time periods, respectively.

*Temperature, salinity, and total alkalinity*:
To isolate the impact of anthropogenic carbon on carbonate chemistry, temperature, salinity, and total alkalinity are assumed to be constant between the historic and modern time periods. It is also

assumed that these parameters are equivalent in the inflowing and outflowing water masses. These values are informed by July averages of observational bottle data at Admiralty Inlet from the Washington Ocean Acidification Center (Supplementary Table 1).

*p*:
The piston velocity, or the gas transfer velocity, is related to wind speed and describes the rate at which air-sea gas exchange occurs. Wind speed in the Strait of Juan de Fuca averages ~11–13 miles per hour, which corresponds to a gas transfer velocity of ~7 m/day[10].

*z*:
The mixed layer depth is assumed to be 100 meters. This value is informed by salinity and temperature profiles from WOAC observational bottle data in the Salish Sea and CTD casts from NANOOS (Northwest Association of Networked Ocean Observing Systems) near Admiralty Inlet.

$\tau$:
The residence time of the Salish Sea is equivalent to the exchange flow rate divided by the box model volume. The volume of our box model, defined by the region between the mouth of the Strait of Juan de Fuca to Admiralty Inlet (where a majority of our corals are located), is 150,000 km x 25 km x 150 m. The exchange flow rate at the mouth of the Strait of Juan de Fuca is estimated to be 140,000 $m^3/s$[11] whereas the exchange flow rate at Admiralty Inlet is estimated to be 10,000–20,000 $m^3/s$[12]. An exchange flow rate representative of our simple box model is calculated by weighting the mouth exchange flow and Admiralty Inlet exchange flow by 2/3 and 1/3, respectively. This yields a weighted residence time of ~65 days.

To account for uncertainties in the calculation of this residence time, we perform a sensitivity analysis at 45, 65, and 85 days.

Results:
We calculate $\Delta pCO_2$ in the Salish Sea from $DIC_{\text{Salish Sea}}$, TA, temperature, and salinity using an independent carbonate chemistry solver in Python. We calculate the $\Delta pCO_2$ at $\tau$ = 65 days and the standard error of the mean of the sensitivity analyses to yield $\Delta pCO_2$ = 185 +/- 15 $\mu$atm. This aligns with the coral-based estimate of $\Delta pCO_2$ in the Salish Sea, suggesting that anthropogenic carbon and thermodynamic chemical equilibrium effects, modulated by estuarine circulation and air-sea gas exchange in the estuary, can explain the 20$^{th}$ century signal.

## 1.3 Comparison between West Coast model and coral records of acidification

Along the West Coast of the US, we directly compare coral estimates with ROMS output, which resolves the coastal CCS. The ROMS resolves carbonate chemistry parameters (Supplementary Fig. 6) and the magnitude of acidification over the 20$^{th}$ century (Methods, Supplementary Fig. 7) at varying depths (Methods, Supplementary Fig. 8).

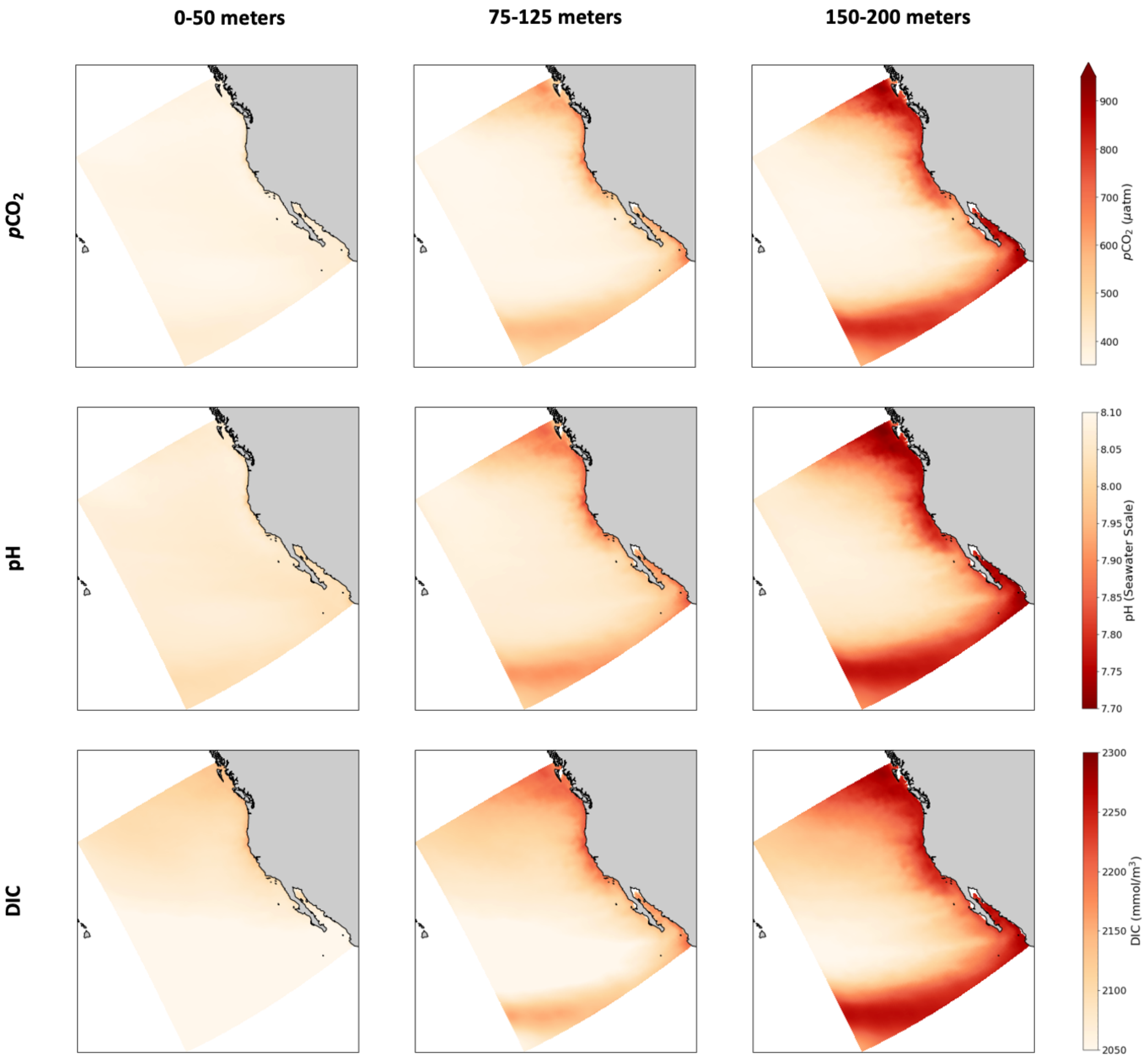


**Supplementary Fig. 6:** Climatological annual mean maps of pH, $pCO_2$, and DIC produced by ROMS. Maps represent 7-year averages (December 2000 – November 2007) at an averaged depth intervals of 0–50 meters, 75–125 meters, and 150–200 meters. Below 50 meters, a band of high-$pCO_2$, low-pH, and high-DIC waters is apparent in the nearshore region. At depth, this pattern of acidification expands in both magnitude and extent into the offshore region. These high $pCO_2$ conditions are expected in this region due to the tilting upward of isopycnals with high amounts of accumulated remineralization products during upwelling.

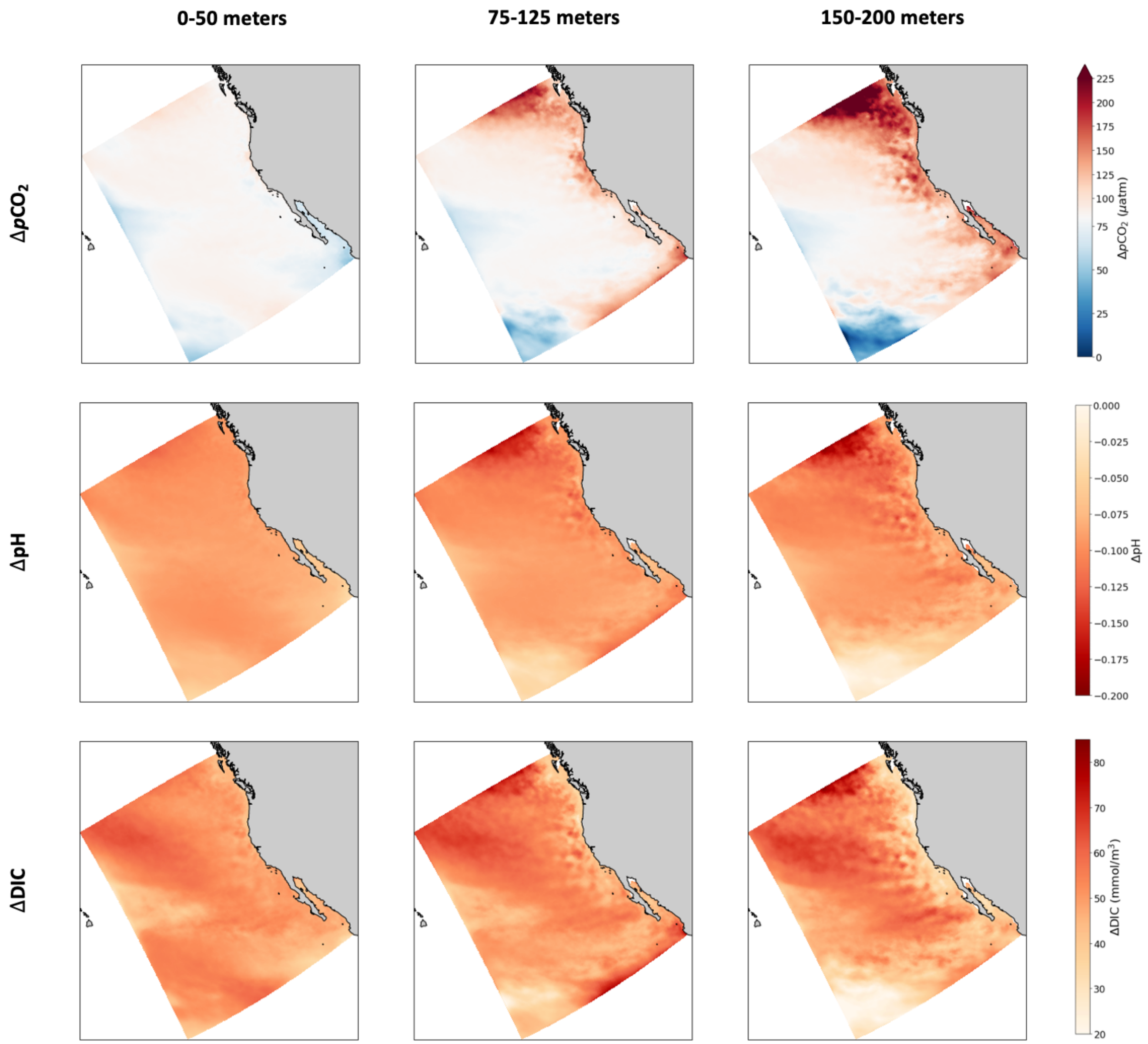


**Supplementary Fig. 7:** Climatological mean maps of 20th century changes in $pCO_2$, pH, and DIC produced by ROMS at 0–50 meters, 75–125 meters, and 150–200 meters. Changes over the past century were evaluated by calculating the difference between a 7-year average in the historic (1897–1904) and modern (2000–2007) model realizations. Below 50 meters depth, we observe amplified acidification in the CCS ($pCO_2$ color bar is centered at atmospheric change in $CO_2$ between 1900 and 2000 = 80 $\mu$atm). Increasing initial DIC in the outcrop region in response to rising atmospheric $CO_2$ results in lower buffering capacity, such that the impact of subsequent remineralization on $pCO_2$ and pH is amplified. We observe relatively muted ΔDIC in the CCS with depth due to older water mass age that has not acquired the recent anthropogenic carbon fingerprint during rapid $CO_2$ emissions[6]. Despite the reduction in ΔDIC at depth in the CCS, we still observe changes in $pCO_2$ that exceed the increase in atmospheric $CO_2$ due to lower buffering capacity and thermodynamic amplification of $pCO_2$ and pH. The southern Gulf of Alaska has experienced the most extreme amplified acidification captured in the model domain over the 20th century.

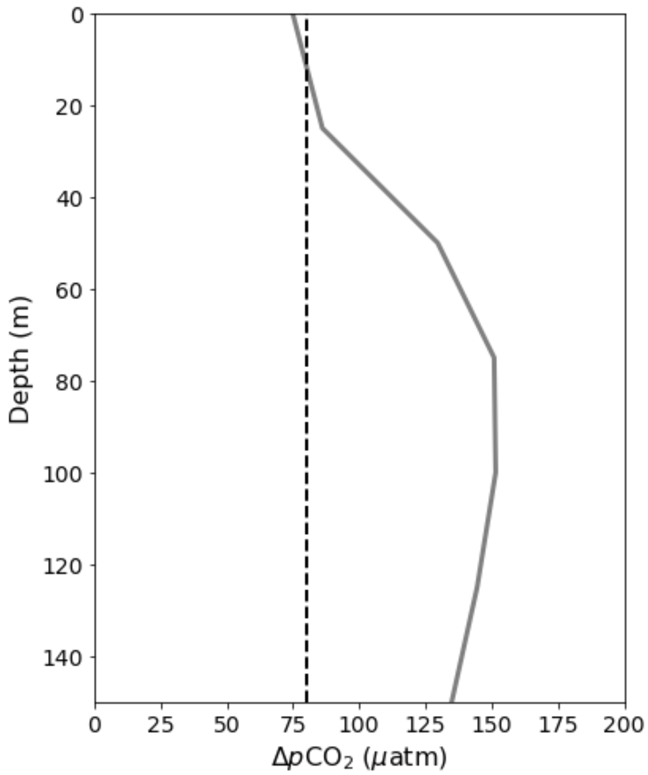


**Supplementary Fig. 8:** Profile of modeled summertime (JJA) $\Delta pCO_2$ in the nearshore CCS (gray). Historic and modern $pCO_2$ are calculated from 7-year averages between November 1897–November 1904 and December 2000–November 2007, respectively. The black dashed line represents the increase in atmospheric $CO_2$ over the 20$^{th}$ century ($\Delta pCO_{2\ atm}$ = 80 $\mu$atm). Data to the right of this dashed line indicates amplified increases in $pCO_2$ with respect to the atmosphere. Below 25 meters depth, the CCS has experienced amplified acidification that increases with depth.

To quantify the magnitude of acidification that can be attributed to changes in upwelling and biological activity, we calculate the fraction of modeled ΔDIC that can be explained by 20$^{th}$ century changes in remineralization (Methods, Supplementary Fig. 9). We then calculate the percent that uptake of anthropogenic $CO_2$ can explain modeled ΔDIC at 0–50 meters, 75–125 meters, and 150–200 meters depth, assuming structured water mass age (Methods. Supplementary Fig. 10).

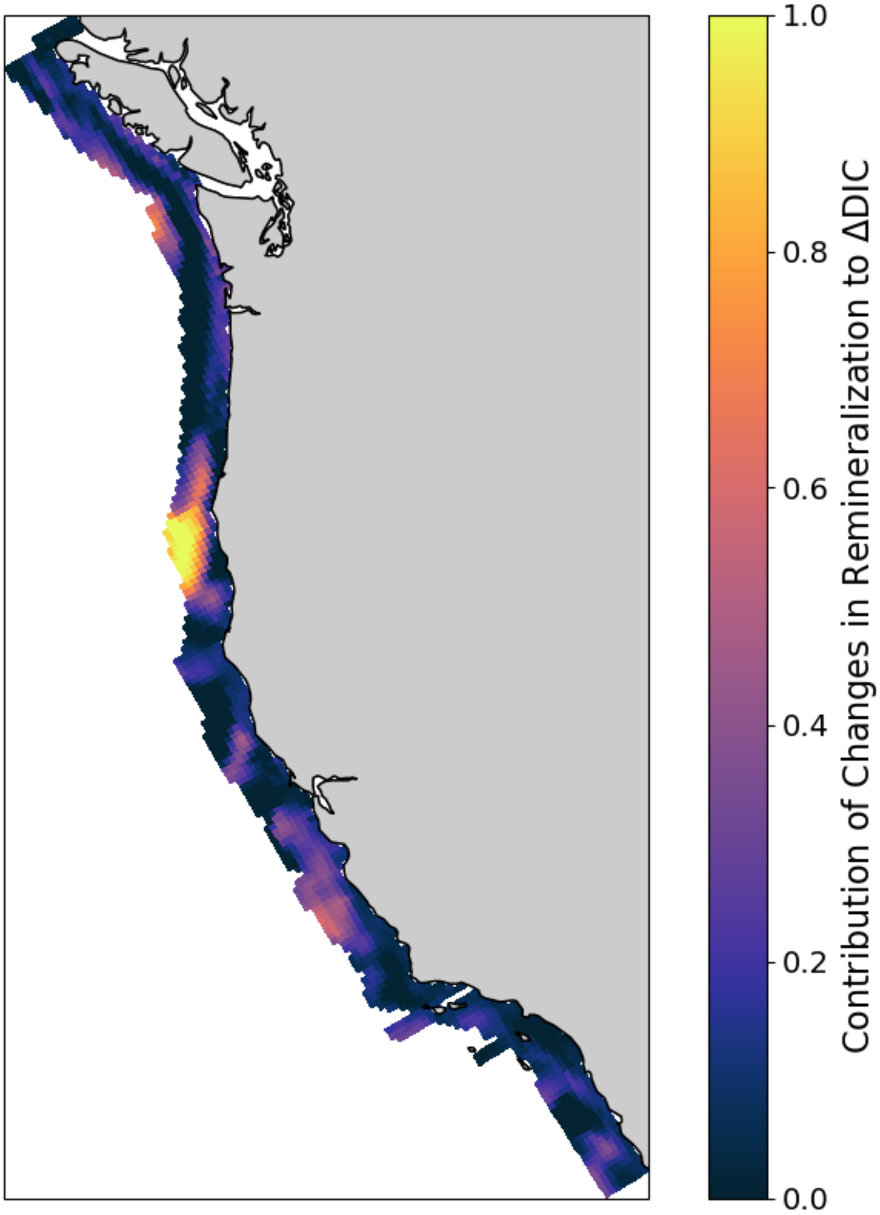


**Supplementary Fig. 9** The ratio of ΔDIC attributed to changes in remineralization to modeled ΔDIC at 0–200 meters depth in the nearshore region that overlaps with the coral locations. Changes in remineralization over the past century are calculated using apparent oxygen utilization and the Redfield ratio determined by Hedges et al. 2002[13] (Methods). 20$^{th}$ century changes in remineralization can account for 15% of modeled ΔDIC at 0–200 meters depth but this process is insufficient to explain most of the observed acidification over the past century.

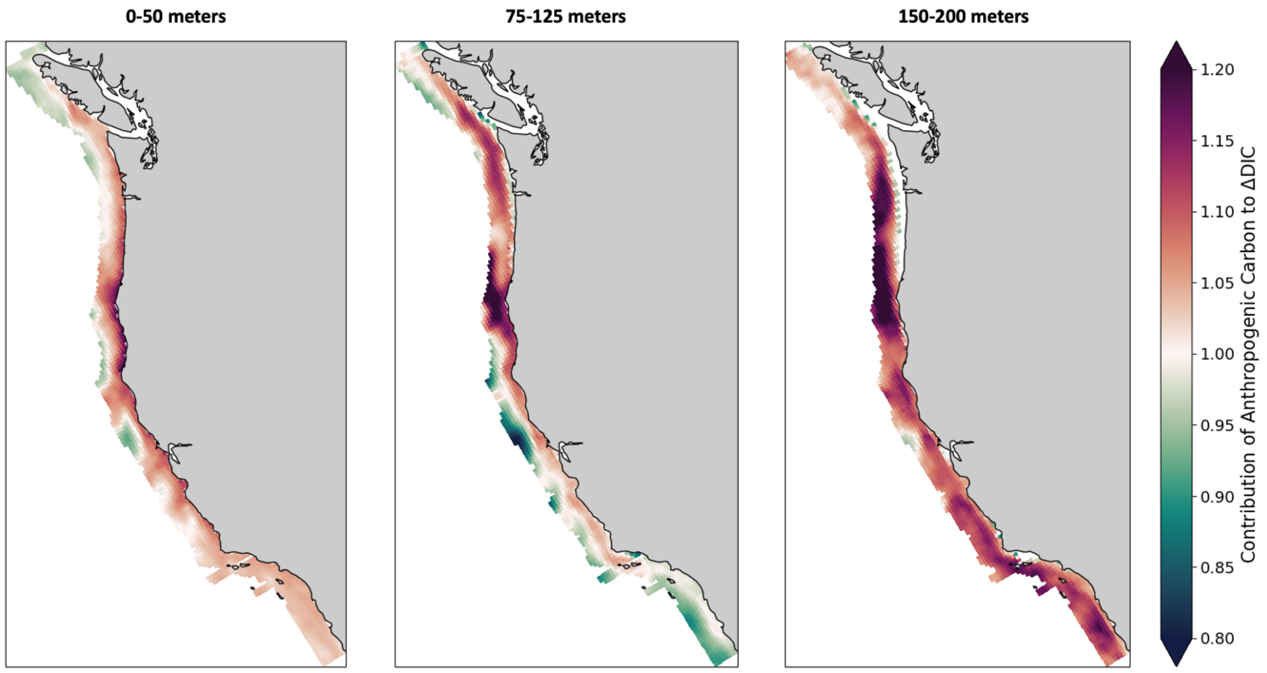


**Supplementary Fig. 10:** The ratio of $\Delta DIC_{preformed}$ attributed to equilibration of atmospheric $CO_2$ (Methods, Equation 3) to modeled ΔDIC at 0–50 meters, 75–125 meters, and 150–200 meters depth ($\Delta DIC_{preformed}/\Delta DIC_{modeled}$, Methods). Each depth bin assumes a different water mass age due to time since last ventilation and contact with the atmosphere (0–50 meters, 75–125 meters, and 150–200 meters depth correspond with 0 years, 13 years, and 25 years old, respectively). Values near 1 indicate that estimated $\Delta DIC_{preformed}$ can fully explain modeled ΔDIC. Averaged over the CCS, estimates of ΔDIC derived from equilibration of atmospheric $CO_2$ at outcropping regions and accompanying buffering effects can explain the modeled ΔDIC within 15% relative error. Remaining minor residuals, identified by the regions shaded white and black, suggest that $CO_2$ invasion alone cannot entirely explain modeled biogeochemical changes over the 20$^{th}$ century, leaving room for second order effects such as shifts in upwelling and biological activity (Supplementary Fig. 9).

Historic model summertime averages of $pCO_2$ are also compared to historic coral $pCO_2$ at every location (Supplementary Fig. 11, Supplementary Table 3). Details about the model- and coral-based estimates of $pCO_2$ can be found in Methods. Our coral record reproduces broad patterns and trends of acidification presented in the model (Figure 2).

While the corals reproduce general patterns of acidification in the model, several locations diverge from historic modeled estimates of $pCO_2$. For instance, while the coral data collected in the Greater Farallones at 50–60 meters depth (USNM 92623 and 92624) are within error of model predictions at these depths, they align better with modeled $pCO_2$ in the upper 25 meters (Figure 2). This difference may result from the inability of the model to resolve key bathymetric features at this site; some evidence suggests that there is a small seamount in this location, however the model resolution is not high enough to capture this variability. In addition, corals at site USNM 78638 were collected from a tidepool where the high variability associated with the intertidal zone likely

influenced the $\delta^{11}$B skeletal signatures. Lastly, our coral data from the Santa Barbara Basin (USNM 92622) deviate most from the model and correspond to a much lower historic $pCO_2$ than that presented in the model. This divergence has been found in another proxy record from the Santa Barbara Basin[14] (Supplementary Discussion 1.4). Although our data from this region are too sparse to be conclusive, the model may fail to capture important regional processes that amplify acidification in the Santa Barbara Basin even more than the rest of the CCS.

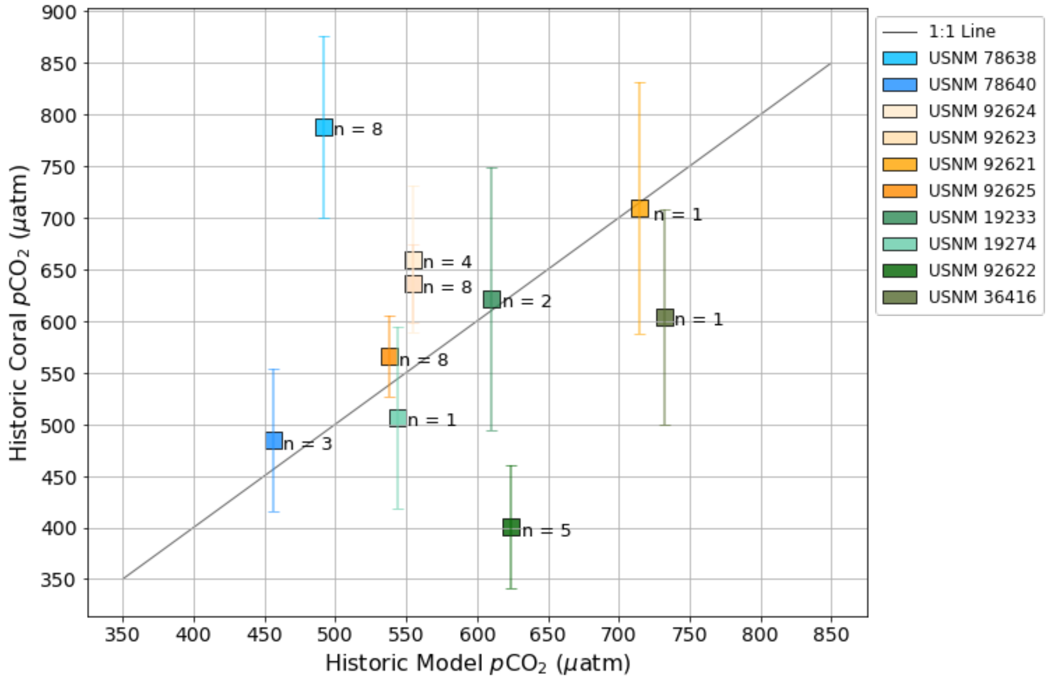


**Supplementary Fig. 11:** Comparison between historic model and coral $pCO_2$ along the West Coast. *Coral data*: $pCO_2$ is calculated from the $\delta^{11}$B-pH relationship at each location (Methods, Supplementary Data). Vertical error bars represent standard errors of the mean. Error bars for locations with only one coral represent the population standard deviation, inferred by locations with 8+ samples. *n* = number of corals sampled at each location. *Model data*: Modeled $pCO_2$ represents a 7-year historic summer average aligning with the peak upwelling season (Supplementary Methods 1.3) over a 36 km x 36 km area centered at the coral location from the nearest depth bin of sample collection (Methods).

**Supplementary Table 3:** Historic West Coast coral $pCO_2$ and pH values (total scale) informed by skeletal $\delta^{11}B$. Average pH values at each location are calculated from the $\delta^{11}B$-pH relationship (Methods, Equation 2) and modeled historic DIC (Methods, Equation 1). The -0.10-offset determined in the Salish Sea is applied to the average pH data (Methods). The average DIC represents modeled, historic summertime DIC in the nearshore CCS at 0–125 meters depth between 1897 and 1904.

| CatalogID | Average DIC CCS ($mmol/m^3$) | Average pH (Offset Applied) | Average $pCO_2$ ($\mu atm$) |
|---|---|---|---|
| USNM 92621 | 2145 | 7.79 | 715 |
| USNM 36416 | 2145 | 7.86 | 608 |
| USNM 92625 | 2145 | 7.89 | 570 |
| USNM 19233 | 2145 | 7.85 | 626 |
| USNM 92624 | 2145 | 7.83 | 664 |
| USNM 92623 | 2145 | 7.84 | 640 |
| USNM 78638 | 2145 | 7.75 | 794 |
| USNM 78640 | 2145 | 7.96 | 488 |
| USNM 92622 | 2145 | 8.03 | 403 |
| USNM 19274 | 2145 | 7.94 | 510 |

1.4 Reconstruction of fossil foraminifera pH and $pCO_2$ based on *Globigerina bulloides* shell weights

We investigate other published proxy records of acidification in the CCS to assess the robustness of our conclusions drawn from the model and coral records. Osborne et al. 2020[14] used the weight and size of fossil foraminifera tests as a proxy for carbonate ion $[CO_3^{2-}]$ from 1897 to 2002 in the Santa Barbara Basin. To directly compare to our coral data, we recast the foraminifera carbonate ion record into pH and $pCO_2$ space. The master variables selected to constrain the carbonate system were 1) $[CO_3^{2-}]$ determined from area-normalized shell weights (ANSW) of *Globigerina bulloides*, as described in Osborne et al. 2020[14] (Supplementary Fig. 12a); and 2) TA inferred from an empirical salinity-TA relationship based on Scripps Pier data spanning 1916-present, as described in Osborne et al. 2020[14] (Supplementary Fig. 12b). Historical temperature and salinity data published in Osborne et al. 2020[14] serve as additional constraints on the pH and $pCO_2$ calculations. From these data we calculated pH and $pCO_2$.

The fossil foraminifera record from the SBB produces acidification signals that outpace modeled signals, suggesting even more amplified acidification. Interestingly, our coral data from the Channel Islands near the SBB (Supplementary Fig. 1) also indicate low historic $pCO_2$ that diverge from modeled estimates of 20$^{th}$ century acidification. Although our data from this region are too sparse to be conclusive, regional processes that may not be resolved in ROMS appear to influence acidification in the SBB even more than the broader and warrant further investigation.

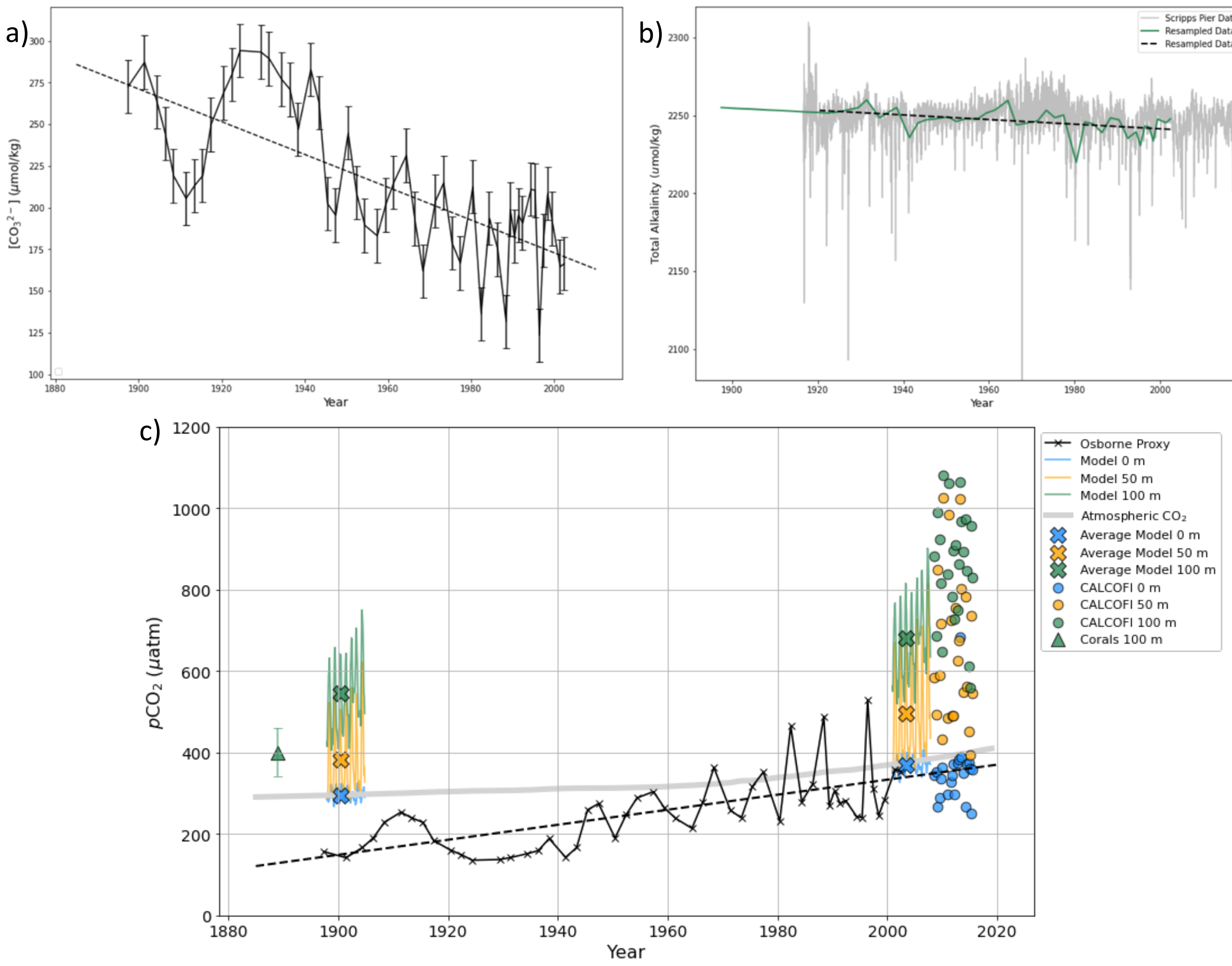


**Supplementary Fig. 12**: a) Reproduced $[CO_3^{2-}]$ data calculated from *G. bulloides* area-normalized shell weights (ANSW) in the Santa Barbara Basin from Osborne et al. 2020[14]. Error bars represent the standard error of the $[CO_3^{2-}]$ mean based on the ANSW-carbonate ion relationship ($\pm$15.63 µmol/kg). b) Derived alkalinity from Scripps Pier salinity data (gray) using an empirical salinity-TA relationship[14]. The green line represents re-sampled TA data to temporally align with the $[CO_3^{2-}]$ record. A best fit line (dashed black) was applied to the re-sampled TA data to extend the record back in time to overlap with the $[CO_3^{2-}]$ record, as described in Osborne et al. 2020[14]. The above $[CO_3^{2-}]$ and TA data represent the master input variables we utilized to calculate pH and $pCO_2$ from the fossil foraminifera proxy record. c) $pCO_2$ time series in the SBB from model simulations, observational data, foraminifera proxy record, and coral proxy records. Model data are represented by colored time series and average values (x's) in the historic and modern simulations at 0, 50, and 100 meters depth. Observational data from the California Cooperative Oceanic Fisheries Investigations (CalCOFI) are represented by the circle markers at 0, 50, and 100 meters depth. $pCO_2$ data informed by the Osborne foraminifera (which typically live in the upper 25 meters of the water column) record is shown by the solid black line and dashed linear trend line). Our coral data from the SBB are represented by the green triangle (collected near 100 meters depth). The error bars represent the standard error of the mean. Atmospheric $CO_2$ is shown by the gray line. Both the coral record and foraminifera records imply stronger acidification than that implied by the model at their respective depths, suggesting that important second-order effects in the SBB may further amplify acidification.

## 2. Large-Scale Climate Factors and the Imprint on our Coral Record

2.1 Impact of the Pacific Decadal Oscillation and climate variability on 20$^{th}$ century acidification

While our coral records suggest that anthropogenic sources act as the dominant driver of 20$^{th}$ century acidification in the CCS, natural large-scale climate patterns also play a role in determining carbonate chemistry. The Pacific Decadal Oscillation (PDO), for instance, oscillates on low-frequency decadal to sub-decadal timescales and influences the strength of coastal upwelling. During positive phases of the PDO, the Aleutian Low pressure cell migrates south resulting in weaker winds and a decrease in upwelling strength[15]. Conversely, negative phases of the PDO result in a strengthening of coastal upwelling. Due to this natural oscillation of upwelling strength on decadal timescales, this signal may be imprinted on the 20$^{th}$ century changes implied by the coral records.

To determine the impact of the PDO on the coral record, we examine the PDO index during the dates of collection for the historic corals, modern corals, and modern model. Monthly PDO indices are based on NOAA's ERSST reconstruction of SSTs and obtained from the National Centers for Environmental Information database. Supplementary Table 4 shows 7–8 year averages of the PDO index during each time period. Since the historic model was forced with climate anomalies from the modern model, the PDO index is equivalent in both hindcast simulations and does not influence the modeled 20$^{th}$ century signal.

To remove the potential influence of the PDO on the 20$^{th}$ century carbonate chemistry signal, it would be ideal to compare records from the historic and modern time periods with similar PDO indices. Along the US West Coast, the average PDO index of the historic corals is within error of that of the modern and historic model (Supplementary Table 4); therefore, the magnitude of change implied by the outer West Coast coral record should be independent of the PDO.

In the Salish Sea, the modern corals were collected during a weak negative phase of the PDO whereas the historic corals were collected during a more extreme negative phase (Supplementary Table 4). While the misalignment of PDO phases across time periods may influence the 20$^{th}$ century signal, this shift in phases actually enhances our conclusions drawn from the data. More negative PDO phases correlate with a strengthening of coastal upwelling; this implies that the historic corals experienced relatively *stronger* upwelling conditions as a result of the PDO compared to the modern corals. This suggests that the historic baseline of acidification may have been relatively high due to the more extreme negative PDO phase. As a result, we might expect this misalignment of PDO indices across eras to reduce the magnitude of $\Delta pCO_2$ or $\Delta$pH implied by the coral record. Instead, we still see a signal that suggests amplified acidification occurred in the Salish Sea between the 1890s and 2020.

**Supplementary Table 4:** Average PDO Index for time periods overlapping with historic coral, modern coral, and modern model records. The time period lengths were inferred by dates of collection and an average coral lifespan of ~8 years. Average PDO indices are inferred from monthly indices from NOAA's ERSST reconstruction of SSTs in the National Centers for Environmental Information database.

| Data Source | Time Period | Average PDO Index | Standard Error of Mean |
|---|---|---|---|
| Historic Corals - West Coast | January 1883 - December 1890 | -0.30 | 0.12 |
| Historic Corals - Salish Sea | January 1887 - December 1894 | -0.80 | 0.13 |
| Modern Corals - Salish Sea | November 2012 - October 2020 | -0.14 | 0.09 |
| Modern Model | December 2000 - November 2007 | -0.38 | 0.09 |

**Supplementary Notes**

## 1. Background Information on Study Area

### 1.1 The California Current upwelling system

The California Current System (CCS), the eastern boundary current off the west coast of North America, spans from British Columbia to Baja California and flows over the continental shelf with a width of 50–100 km[16]. Eastern boundary current systems experience low pH and high partial pressure of $CO_2$ ($pCO_2$) because of upwelling and ocean circulation[17,18]. As a result, eastern boundary current systems naturally contain some of the most acidified waters in the ocean.

It is hypothesized that climate change will cause a strengthening of alongshore winds due to sharper land-sea temperature gradients, consequently increasing the frequency and intensity of upwelling events in eastern boundary current systems and leading to higher overall acidification levels[19]. While successive analyses indicate that upwelling will intensify in the poleward regions of eastern boundary currents[20], others have found muted responses in the CCS compared to other eastern boundary currents[21,22] and suggest that the anthropogenic impact on upwelling strength in the CCS will not exceed natural variability until the end of the 21st century[23]. Still others have analyzed historical wind data and model re-analyses over the past two to six decades and found a strengthening of upwelling-favorable winds[24–26]. The wide spread of results in the literature underscores the unknown role dynamic climate effects will play in a changing CCS. Due to the complexity of processes that impact biogeochemistry in the CCS and Salish Sea, a central question is whether acidification in vital and productive eastern boundary current systems such as the CCS will follow the pace of increasing atmospheric $CO_2$, or if dynamical climate effects or other processes will act to either amplify or attenuate acidification.

### 1.2 Characterizing the carbonate chemistry in the Salish Sea

The CCS has a strong influence on coastal ecosystems along the US West Coast and connected water bodies[1,12] (Hickey et al. 1989; Hickey 1979). For instance, the Salish Sea is a marginal sea of the North Pacific that encompasses the coastal waterways of southern British Columbia, Canada and northwestern Washington, US, including Puget Sound. Forming a transition zone between the open ocean and coast, the Salish Sea is one of the largest estuaries in the US of economic and cultural importance, and represents a living lab where complex climate, oceanic, riverine, and land-based processes converge[27] (Stewart et al. 2017). Due to the mixing of upwelled source waters, the carbonate chemistry of the Salish Sea also serves as a useful indicator of upwelling dynamics in the broader CCS[28] (Feely et al. 2010).

In addition to the influence of the CCS, seasonal variability in the Salish Sea impacts carbonate chemistry and provides an important constraint on the main driver of 20th century acidification. Seasonal variability of the carbonate system is influenced by biological activity, influx of freshwater, and mixing of upwelled bottom-water from the CCS[29,30]. Given that attribution of individual effects is difficult, questions remain regarding the dominant force responsible for seasonal variation. We set out to determine whether 1) biological processes or 2) mixing of upwelled and riverine source waters primarily controls interannual variability in the carbonate

system. Our analyses focus on characterizing seasonal variation at depth due to the benthic nature of the corals. We find that seasonal shifts in the mixing ratio between upwelled CCS water and Salish Sea water, which is also influenced by rivers, dominates the variability in carbonate system parameters in the modern system.

*Influence of rivers on Salish Sea carbonate chemistry*

Admiralty Inlet, situated at the interface between the Strait of Juan de Fuca and Puget Sound, serves as a useful case study due to the breadth of Washington Ocean Acidification Center (WOAC) bottle data and historic and modern coral data. Admiralty Inlet represents a tidally well-mixed area that experiences saltwater influence from the CCS and, like the rest of the Salish Sea, freshwater influence from rivers. The Skagit River represents the largest freshwater input to Puget Sound[31,32] and feeds into Skagit Bay with an average flow rate of ~17,000 ft$^3$/sec (USGS Skagit River gauge 12200500). Skagit River discharge varies seasonally and peaks in the early summer and winter months, coinciding with rapid snowpack melt and maximum precipitation, respectively (USGS Skagit River gauge 12200500).

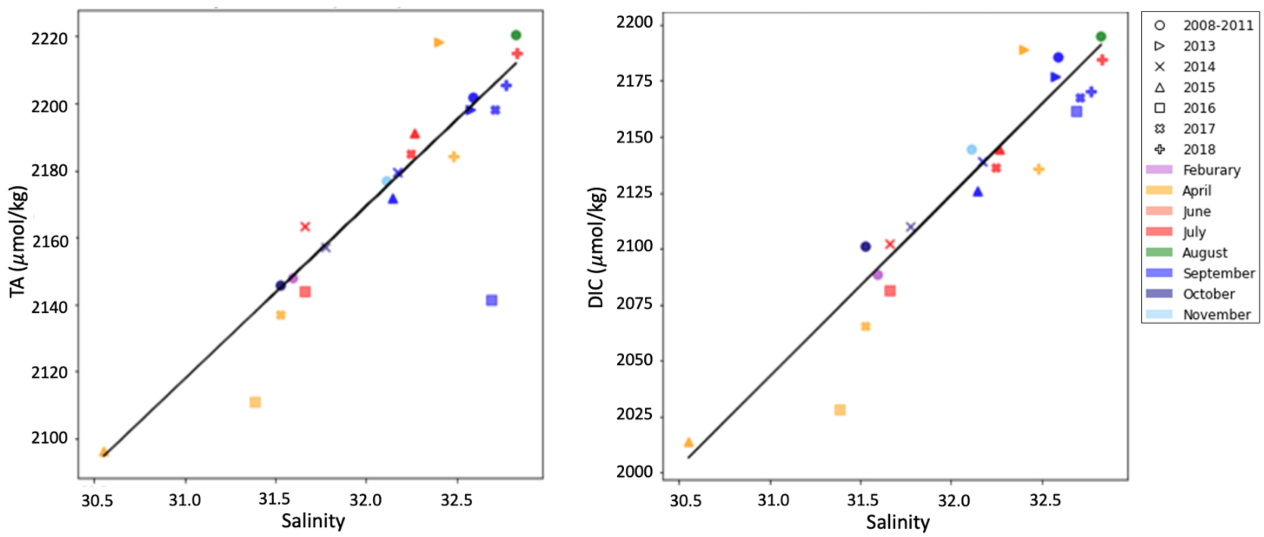


**Supplementary Fig. 13:** Relationships between a) total alkalinity (TA) and salinity and b) DIC and salinity from WOAC Station 21 near Admiralty Inlet at 70 meters that align with the coral depth of collection. Unsurprisingly, both TA and DIC correlate strongly with salinity. In general, salinity is lower in the late spring and early summer months (April–June) compared with the fall (September–November).

*Determining the dominant control on seasonal variability*

Biological and mixing processes are tested as the dominant control on seasonal variability of carbonate chemistry in the Salish Sea. To investigate each mechanism, we invoke process-based equations to draw biological activity and mixing lines onto a total alkalinity (TA) vs DIC plot (Supplement Figure 1.2). The mixing line is based on a two end-member mixing scenario between Salish Sea waters and summer upwelled waters from the CCS. Average properties for the Salish Sea were obtained from WOAC bottle data at Admiralty Inlet (Supplementary Methods 1.2) and average properties of upwelled water were obtained from a Regional Ocean Modeling System

(ROMS) that resolves the CCS at 150–200 meters depth at the mouth of the Strait of Juan de Fuca. The properties of the upwelled waters from the model align with published properties of the core CCS off the Washington coast[9,33]. The process line that represents primary production and remineralization (biological activity) was generated from the DIC:TA values implied by Redfield stoichiometry. Pelletier et al. 2018[34] found tight coupling between biogeochemical changes induced by the phytoplankton growing season in Puget Sound.

Supplementary Fig. 14a shows that the slope of the observational data aligns more closely with the slope of the mixing line compared to the biological activity line. Furthermore, normalizing the TA and DIC for salinity causes the data to collapse and a majority of the seasonal variability is removed (Supplementary Fig. 14b). Although biological arguments may be invoked to explain a portion of the observational data, salinity-based processes are the dominant mechanism responsible for the observed seasonality in carbonate chemistry. This foundational understanding of carbonate chemistry variability provides important context to consider shifts in source water mixing as the driving force of 20th century acidification in the Salish Sea, described in Supplementary Discussion 1.

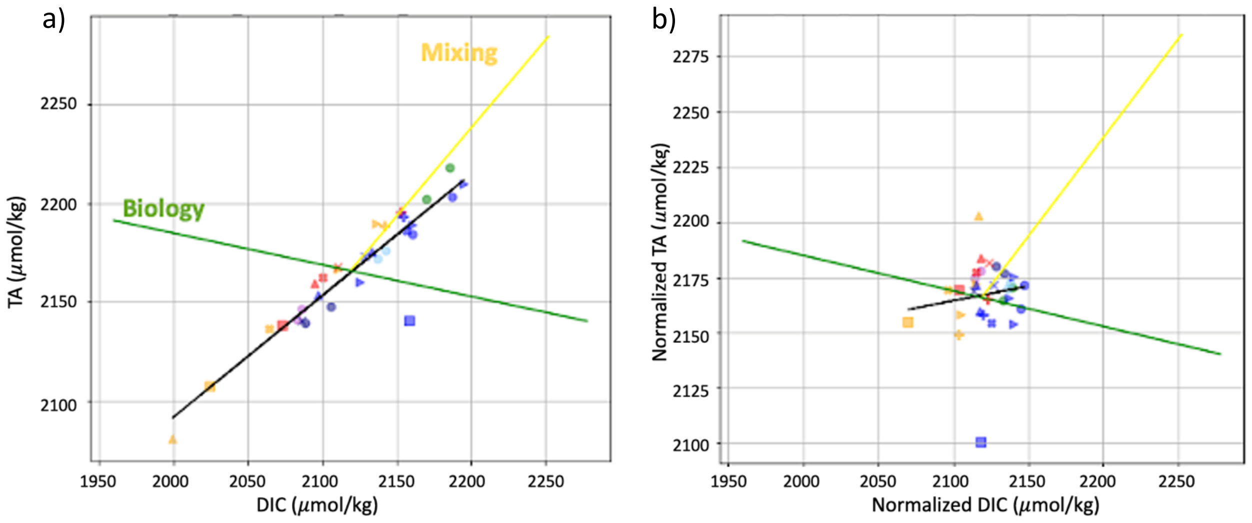


**Supplementary Fig. 14:** TA vs DIC plots of observational data from WOAC station 21 at 70 meters depth provide information about drivers of seasonal variability in carbonate chemistry. Process lines depicting primary production (green) and mixing (yellow) are produced alongside a best fit line (black) of the observational data. a) The mixing line follows the observational data more closely than the biological process line, suggesting that mixing plays a larger role in controlling seasonal variability in the Salish Sea. b) Upon normalizing the TA and DIC data for salinity, the trend in the observational data disappears. This suggests that the driver of seasonal variability in the Salish Sea is salinity-based, further indicating mixing processes.

**Supplementary References**


1. Gagnon, A. C., Gothmann, A. M., Branson, O., Rae, J. W. B. & Stewart, J. A. Controls on boron isotopes in a cold-water coral and the cost of resilience to ocean acidification. *Earth and Planetary Science Letters* 554, 116662 (2021).
2. Fadlallah, Y. Population dynamics and life history of a solitary coral, Balanophyllia elegans, from Central California | Oecologia. https://link.springer.com/article/10.1007/BF00399217 (1983).
3. Jantzen, C. *et al.* In situ short-term growth rates of a cold-water coral. *Marine and Freshwater Research* 64, 631–641 (2013).
4. Beck, K. K. Seasonal growth and skeletal composition of the cold-water coral Desmophyllum dianthus. (2018).
5. Alin, S. R. *et al.* A compiled data product of profile, discrete biogeochemical measurements from 35 individual cruise datasets collected from a variety of ships in the southern Salish Sea and northern California Current System (Washington state marine waters) from 2008-02-04 to 2018-10-19 (NCEI Accession 0238424). NOAA National Centers for Environmental Information https://doi.org/10.25921/ZGK5-EP63 (2021).
6. Feely, R. A. *et al.* Chemical and biological impacts of ocean acidification along the west coast of North America. *Estuarine, Coastal and Shelf Science* 183, 260–270 (2016).
7. Kroeker, K. J. *et al.* Impacts of ocean acidification on marine organisms: quantifying sensitivities and interaction with warming. *Global Change Biology* 19, 1884–1896 (2013).
8. Johannessen, S. C., Macdonald, R. W. & Strivens, J. E. Has primary production declined in the Salish Sea? *Can. J. Fish. Aquat. Sci.* 78, 312–321 (2021).
9. Murray, J. W. *et al.* An inland sea high nitrate-low chlorophyll (HNLC) region with naturally high pCO $_2$: An HNLC inland sea with high pCO $_2$. *Limnol. Oceanogr.* 60, 957–966 (2015).
10. Donelan, M. A. & Soloviev, A. V. A mixing length model for the aqueous boundary layer including the effect of wave breaking on enhancing gas transfer. *IOP Conf. Ser.: Earth Environ. Sci.* 35, 012001 (2016).
11. MacCready, P. *et al.* Estuarine Circulation, Mixing, and Residence Times in the Salish Sea. *Journal of Geophysical Research: Oceans* 126, e2020JC016738 (2021).
12. Sutherland, D. A., MacCready, P., Banas, N. S. & Smedstad, L. F. A Model Study of the Salish Sea Estuarine Circulation. *Journal of Physical Oceanography* 41, 1125–1143 (2011).
13. Hedges, J. I. *et al.* The biochemical and elemental compositions of marine plankton: A NMR perspective. *Marine Chemistry* 78, 47–63 (2002).
14. Osborne, E. B., Thunell, R. C., Gruber, N., Feely, R. A. & Benitez-Nelson, C. R. Decadal variability in twentieth-century ocean acidification in the California Current Ecosystem. *Nature Geoscience* 13, 43–49 (2020).
15. Mantua, N. J. & Hare, S. R. The Pacific decadal oscillation. *Journal of oceanography* 58, 35–44 (2002).
16. Hickey, B. M. The California current system—hypotheses and facts. *Progress in Oceanography* 8, 191–279 (1979).
17. Chan, F. *et al.* Persistent spatial structuring of coastal ocean acidification in the California Current System. *Sci Rep* 7, 2526 (2017).
18. Reum, J. C. P. *et al.* Interpretation and design of ocean acidification experiments in upwelling systems in the context of carbonate chemistry co-variation with temperature and oxygen. *ICES Journal of Marine Science* 73, 582–595 (2016).

19. Bakun, A. Global Climate Change and Intensification of Coastal Ocean Upwelling. *Science* 247, 198–201 (1990).
20. Rykaczewski, R. R. *et al.* Poleward displacement of coastal upwelling-favorable winds in the ocean's eastern boundary currents through the 21st century. *Geophysical Research Letters* 42, 6424–6431 (2015).
21. Wang, D., Gouhier, T. C., Menge, B. A. & Ganguly, A. R. Intensification and spatial homogenization of coastal upwelling under climate change. *Nature* 518, 390–394 (2015).
22. Howard, E. M. *et al.* Attributing Causes of Future Climate Change in the California Current System With Multimodel Downscaling. *Global Biogeochem. Cycles* 34, (2020).
23. Brady, R. X. On the role of climate modes in modulating the air-sea CO2 fluxes. *Biogeosciences* 16, 329–346 (2019).
24. Sydeman, W. J. *et al.* Climate change and wind intensification in coastal upwelling ecosystems. *Science* 345, 77–80 (2014).
25. Turi, G., Lachkar, Z., Gruber, N. & Münnich, M. Climatic modulation of recent trends in ocean acidification in the California Current System. *Environ. Res. Lett.* 11, 014007 (2016).
26. Quilfen, Y., Shutler, J., Piolle, J.-F. & Autret, E. Recent trends in the wind-driven California current upwelling system. *Remote Sensing of Environment* 261, 112486 (2021).
27. Stewart, H. The Salish Sea: Jewel of the Pacific Northwest. *BC Studies* 205–205 (2017).
28. Feely, R. A. *et al.* The combined effects of ocean acidification, mixing, and respiration on pH and carbonate saturation in an urbanized estuary. *Estuarine, Coastal and Shelf Science* 88, 442–449 (2010).
29. Alin, S. R. *et al.* Evaluating the Evolving Ocean Acidification Risk to Dungeness Crab: Time-Series Observations and Modeling on the Olympic Coast, Washington, Usa. *Oceanography* 36, 138–147 (2023).
30. Alin, S. R. *et al.* A decade-long cruise time series (2008–2018) of physical and biogeochemical conditions in the southern Salish Sea, North America. *Earth System Science Data* 16, 837–865 (2024).
31. Babson, A. L., Kawase ,M. & and MacCready, P. Seasonal and Interannual Variability in the Circulation of Puget Sound, Washington: A Box Model Study. *Atmosphere-Ocean* 44, 29–45 (2006).
32. Cokelet, E. D., Stewart, R. J. & Ebbesmeyer, C. C. The annual mean transport in Puget Sound. (1989).
33. Pierce, S. D., Barth, J. A., Shearman, R. K. & Erofeev, A. Y. Declining Oxygen in the Northeast Pacific*. *Journal of Physical Oceanography* 42, 495–501 (2012).
34. Pelletier, G., Roberts, M., Keyzers, M. & Alin, S. R. Seasonal variation in aragonite saturation in surface waters of Puget Sound – a pilot study. *Elementa: Science of the Anthropocene* 6, 5 (2018).
