# Supplementary material for: A century of change in the California Current: upwelling system amplifies acidification
Source: Nat Commun. 2025 Nov 13;16:9661. doi: 10.1038/s41467-025-63207-6 (PMC12615725; doi:10.1038/s41467-025-63207-6)
Supplement: Supplementary file 3 — Description of Additional Supplementary Files [file 41467_2025_63207_MOESM3_ESM.pdf]

Description of Additional Supplementary Files

File Name: Supplementary Data 1
Description: Historic and modern coral metadata and boron isotope measurements.
